# Vehicle Detection and Attribution from a Multi-Sensor Dataset Using a Rule-Based Approach Combined with Data Fusion

**DOI:** 10.3390/s23218811

**Published:** 2023-10-30

**Authors:** Lindsey A. Bowman, Ram M. Narayanan, Timothy J. Kane, Eliza S. Bradley, Matthew S. Baran

**Affiliations:** 1 Applied Research Laboratory, The Pennsylvania State University, State College, PA 16801, USA; las98@arl.psu.edu (L.A.B.); msb236@arl.psu.edu (M.S.B.); 2 Department of Electrical Engineering, The Pennsylvania State University, University Park, PA 16802, USA; tjk7@psu.edu; 3 Department of Student Affairs Research and Assessment, The Pennsylvania State University, University Park, PA 16802, USA; esb165@psu.edu

**Keywords:** remote sensing, vehicle detection, lidar, satellite imagery, object detection, data fusion, multi-sensor

## Abstract

Vehicle detection using data fusion techniques from overhead platforms (RGB/MSI imagery and LiDAR point clouds) with vector and shape data can be a powerful tool in a variety of fields, including, but not limited to, national security, disaster relief efforts, and traffic monitoring. Knowing the location and number of vehicles in a given area can provide insight into the surrounding activities and patterns of life, as well as support decision-making processes. While researchers have developed many approaches to tackling this problem, few have exploited the multi-data approach with a classical technique. In this paper, a primarily LiDAR-based method supported by RGB/MSI imagery and road network shapefiles has been developed to detect stationary vehicles. The addition of imagery and road networks, when available, offers an improved classification of points from LiDAR data and helps to reduce false positives. Furthermore, detected vehicles can be assigned various 3D, relational, and spectral attributes, as well as height profiles. This method was evaluated on the Houston, TX dataset provided by the IEEE 2018 GRSS Data Fusion Contest, which includes 1476 ground truth vehicles from LiDAR data. On this dataset, the algorithm achieved a 92% precision and 92% recall. It was also evaluated on the Vaihingen, Germany dataset provided by ISPRS, as well as data simulated using an image generation model called DIRSIG. Some known limitations of the algorithm include false positives caused by low vegetation and the inability to detect vehicles (1) in extremely close proximity with high precision and (2) from low-density point clouds.

## 1. Introduction

Object detection research has been increasingly popular for the past several decades. This emphasis is due, in part, to the advancement of machine learning methods. Vehicle detection is of specific interest to a wide range of communities. It spans the gamut from traffic monitoring and control to disaster relief efforts and military applications, including intelligence, surveillance, and reconnaissance (ISR). Historically, vehicle detection has been achieved using overhead imagery. Although these methods have proved successful, there are still several limitations and challenges associated with them, such as limited information and confusion with similar objects. Overhead imagery tends to lack a significant amount of detail for smaller objects, even at high spatial resolutions.

This can lead to a higher false-positive rate, as objects with characteristics similar to those of vehicles are also detected. Three-dimensional data tend to fill this void, as it contains intensity values and the same *x* and *y* spatial information but also includes elevation information in *z*. This additional information can significantly reduce the number of false positives by observing the behavior of points in the *z*-direction. Some 3D products also supply red, green, and blue (RGB) color information. LiDAR point clouds are a specific type of 3D product that can be used for vehicle detection. These point clouds can be used as the sole source of data or in combination with imagery and road networks to improve detection results. This article aims to demonstrate the value of 3D information, in combination with imagery and road networks, for vehicle detection, in complement to other work that has investigated the value of 3D data for remote sensing classification tasks [1].

## 2. Related Works

Vehicle detection algorithms can be separated into three main categories for both technique and sensing modality. Techniques can be classified as model-driven, learning, or classical approaches and typically operate on color imagery, LiDAR point clouds, or a fusion of the two.

Historically, vehicle detection methods have used model-driven and color imagery techniques, such as those described by Hinz et al. and Rajagopalan et al. [2,3,4]. The initial model-driven techniques were based largely on implicit, appearance-based models [4]. These models are derived from exemplar images, with their feature vectors formed from image statistics. Statistical information about the background and counter examples are also captured. To detect the presence of a vehicle within a region, the feature vector for the region is computed and compared to the model. An explicit modeling approach was seen to produce significantly better results in complex urban scenes. Explicit model-driven techniques typically use a 2D box or 3D wire frame model. Adaptive gradients are used across the vehicle based on certain vehicle attributes and scene properties. A tree-like model hierarchy can be used to determine the likelihood that a candidate in the image is a vehicle and provide the best match. A benefit of this algorithm is that very few models are needed to accurately detect vehicles.

Recent work, however, has focused largely on the use of machine learning and deep learning methods to extract vehicles from color imagery or LiDAR data. A substantial number of LiDAR-based publications in this realm utilize ground-based LiDAR systems. This is because many vehicle detection applications are geared towards the rapidly growing research field of self-driving cars [5,6]. Detecting vehicles from airborne LiDAR data is useful for other applications, such as vehicle counting and traffic maps. The Cars Overhead with Context (COWC) approach uses ResCeption, a neural network that combines inception layers with residual learning components [7]. These layers are then stacked and allow the network to detect vehicles within the image, which provides “context”. Deep learning methods, such as the one described above, generally perform fairly well and yield significantly better performance metrics but also require a large amount of training data and are not always robust when evaluated on an unfamiliar scene [8]. To work around this issue, the authors used a specific transfer learning technique called domain adaptation (DA). Using this technique removes the need for labeled training data in the target domain but only slightly improves the results. Deep learning methods can also be applied to 3D point clouds. VoxelNet [9] is a generic 3D object detection framework that generates 3D bounding boxes directly from raw point clouds without manual feature engineering. The framework first processes the point cloud by dividing it into uniformly sized voxels. Each voxel is then represented as a unified feature through a voxel feature encoding layer (VFE). This is then input into the Region Proposal Network (RPN), which outputs the classification probability and 3D bounding box. VoxelNet reported state-of-the-art performance measures across datasets of varying difficulty. VoxelNet is designed to use ground-based LiDAR point clouds; however, unofficial implementations have been adapted to use airborne LiDAR with similar success.

Vehicular synthetic aperture radar (SAR) has been demonstrated as a promising technique to augment radar imaging capability by exploiting the vehicle motion to provide 2D and 3D images of the surroundings. SAR has higher resolution compared to standard automotive radars, provided that the motion is precisely known [10]. The potentials in SAR imaging were subsequently analyzed by evaluating the implications of coherent data processing over a significantly longer integration time than allowed by conventional radar imaging [11]. This suggests the possibility of generating high-resolution mapping of urban (or extra-urban) environments by the application of synthetic aperture radar (SAR) processing concepts to the data collected by mm-wave automotive radars installed onboard commercial vehicles. A novel rectangle-invariant rotatable convolution (RIRConv) technique was developed to accurately determine the convolutional sampling locations for vehicle targets. The research exploited the shape characteristic of vehicle targets in SAR images, which always retain a rectangular shape despite their varying sizes, aspect ratios, and rotation angles [12].

Although multimodal fusion frameworks have a great many benefits, few have exploited them within recent years [13]. Multi-modal fusion frameworks have only recently started to gain attention. Recent work has shown that there is great benefit from fusing information gathered from different sensing modalities. Building upon the VoxelNet framework, SEGVoxelNet [14] uses an image segmentation framework in combination with an improved implementation of VoxelNet to detect vehicles from RGB images and LiDAR point clouds. When evaluated on the KITTI 3D vehicle detection benchmark dataset, the authors showed that SEG-VoxelNet outperformed state-of-the-art methods. The work by Liu et al. also took a data fusion approach with co-registered RGB imagery and LiDAR point cloud data [15]. The authors used Gaussian process (GP) classification to generate an initial vehicle probability map, which used training data. A gradient-based segmentation (GSEG) algorithm was then applied to the digital surface model (DSM) generated from the LiDAR data, followed by a region growing process. The initial probability map was then refined based on certain physical properties, such as size and surface shape. The results presented by Liu et al. showed that this method achieved 90.8% precision and 93.7% recall.

A U-Net based CNN with feature covariance loss and self-training process model developed was first trained in the source domain with the ISPRS 2-D semantic labeling contest dataset. The pretrained model was then applied on the IEEE GRSS dataset with feature covariance loss to alleviate the domain shift, and the segmentation results demonstrated effective land cover classification without annotations [16]. Methods for automatic 3D urban building modeling typically employ multi-view image inputs to recover point clouds and 3D models of buildings. An efficient digital surface model (DSM) estimation-driven reconstruction framework was developed that was able to reconstruct 3D building models from single-view remote sensing images as inputs [17]. A two-stage automatic hierarchical clustering method was presented for accurate building segmentation from LiDAR point clouds using Gaussian mapping to identify several types of building geometric shapes and parameterizing these to complete the point cloud’s coarse segmentation. Then, a binary integer programming optimization model was used to reassign the outlier points to complete the fine segmentation [18]. To implement unsupervised domain adaptation (UDA) in ship classification, i.e., to transfer a machine learning model from a labeled source domain to an unlabeled target domain, employing combinations of semi-supervised learning techniques with standalone UDA approaches was investigated. The effectiveness of combining recent semi-supervised learning methods with other domain adaptation algorithms for the purpose of improving the robustness of ship classifiers in maritime satellite imagery was demonstrated [19].

Even fewer have explored classical methods. The approaches mentioned above are similar in the fact they all require some amount of training data. Often times, with machine and deep learning approaches, the algorithm is not extremely robust for scene types other than those similar to the training data. Even less so than multi-modal fusion frameworks, classical approaches are not widely popular. Classical approaches are those that do not make use of any training data or learning components at any point in the pipeline. One example of a classical approach uses an object-based point cloud analysis method [20]. Using solely LiDAR data, the authors first locate ground points using a Triangular Irregular Network (TIN) and identify potential vehicle points based on their height above the terrain. Connected components analysis is then used to cluster the potential vehicle points. Finally, the physical properties (area, rectangularity, and elongation) of the cluster are used to determine which clusters are vehicles. This method was tested on an urban scene at three different point densities. At a density of 40 ppsm, the algorithm achieved 80.1% precision and 84.5% recall. This decreased to 77.2% precision and 80.2% recall for a point density of 20 ppsm and 73.7% precision and 65.6% recall for a density of 10 ppsm.

Therefore, there appears to be a void in vehicle detection algorithms that take a classical approach using multi-modal data. The approach presented in this paper aims to avoid the need for training data, as this can be computationally expensive and not robust for scene variations. It also aims to incorporate the benefits of using data from multiple sensing modalities. The three main components include point cloud classification, vehicle detection using rule-based and Laplacian of Gaussian methods, and vehicle attribution.

## 3. Study Areas and Datasets

We evaluated a classical approach for vehicle detection on three datasets. We refer to these datasets as “IEEE” for data from the 2018 IEEE GRSS Data Fusion Contest [21], “ISPRS” for data from the International Society for Photogrammetry and Remote Sensing [22], and “DIRSIG” for data generated from the Digital Imaging and Remote Sensing Image Generation model [23].

### 3.1. The 2018 IEEE GRSS Dataset

**Figure 1:**
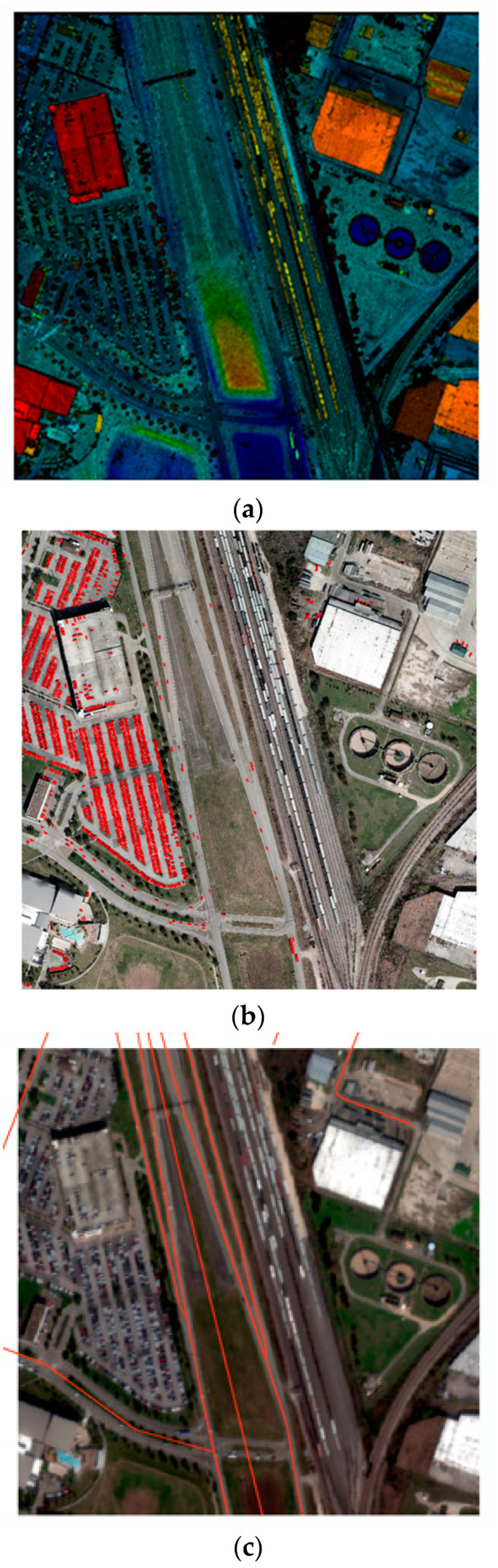
Data for the tile chosen from the 2018 IEEE GRSS Data Fusion Contest: (**a**) LiDAR point cloud, overhead view; (**b**) RGB imagery with ground truth (red); (**c**) HSI imagery with road network (orange).

The IEEE dataset included co-collected multispectral-LiDAR, hyperspectral imagery, and high-resolution RGB imagery. The data were captured from an airborne platform at an altitude of 500 m, which gave a top-down view of cars in the imagery, as opposed to some other vehicle detection datasets that provide more of an angled view. The LiDAR data were collected using an Optech Titan MW at wavelengths of 1550 nm, 1064 nm, and 532 nm, with a point density of approximately 33 points per square meter (ppsm). The hyperspectral imagery was collected with the ITRES CASI 1500 and contained 48 bands from 380–1050 nm at 1 m resolution, while the RGB imagery were collected using a DiMAC ULTRALIGHT+ at 5 cm resolution. This dataset contained multiple tiles roughly 600 m × 600 m. The tile chosen for this analysis contained 1476 vehicles and included a variety of features and challenges, such as a parking garage with vehicles located on the top level, parking lots with closely spaced vehicles, and vehicles on the roads, all of which increased the difficulty in detecting and separating vehicles. Additional features included typical urban objects, power lines, buildings, vegetation, and terrain features. The 1476 ground truth vehicle polygons were created by hand from primarily RGB imagery and supplemented by LiDAR data. Road networks were readily available for download in the form of shapefiles from a variety of sources. Because of this, they lent themselves well as supplemental data to the feature extraction. We obtained road network shapefiles from OpenStreetMap (OSM) and clipped them to the extent of the tile. The contents of this dataset are shown in Figure 1.

The algorithm only requires a point cloud as input; however, additional data, such as roads and imagery, leads to a better detection map. The results presented in this paper, pertaining to the IEEE dataset, were the result of point clouds, RGB imagery, HSI imagery, and road networks as inputs.

### 3.2. The ISPRS Benchmark Dataset

**Figure 2:**
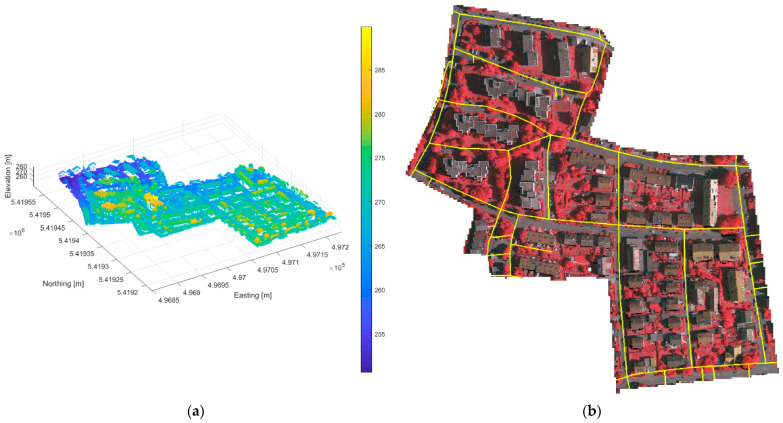
Data from the ISPRS benchmark on urban classification and 3D building reconstruction: (**a**) LiDAR point cloud; (**b**) NRG image with road network (yellow).

The ISPRS dataset was the result of an effort to provide researchers access to datasets for urban classification and reconstruction. The dataset, captured over Vaihingen, Germany, is comprised of aerial imagery and LiDAR data, which can be seen in Figure 2. The imagery was collected from an altitude of 900 m with Intergraph’s Z/I Imaging Digital Mapping Camera (DMC) at a spatial resolution of 8 cm. The LiDAR data were acquired by a Leica ALS50 system at an altitude of 500 m with an average point density of 4 ppsm and contained three bands corresponding to the near-infrared (NIR), red, and green channels (NRG) [24]. It contains several types of study areas, including an inner city, high rises, and residential neighborhoods. Unlike the IEEE dataset, the imagery and LiDAR data for the ISPRS benchmark were not co-collected. Imagery that was not co-collected was still extremely valuable for excluding false positives due to vegetation; however, it could not be used to confirm the presence of true vehicles, as the co-collected data could. Compared to the IEEE dataset, the ISPRS dataset has a low point density, meaning that there are fewer samples on any given target. This can pose a challenge for object detection algorithms, as it becomes harder to distinguish objects and identify one object class from another. In addition to the challenges presented by a low-density point cloud, tree overhang can sometimes occlude or partially occlude vehicles parked along the roadside or in driveways. Similar to the IEEE dataset, road networks were obtained and clipped to the extent of the scene.

The ISPRS dataset contains several types of study areas, including an inner city, high rises, and residential neighborhoods. The area chosen to evaluate the vehicle detection algorithm was selected based on the available ground truth and covered both high rises and residential buildings. A small subset of the data contains per point classification labels, including those for vehicles. This scene differs from the IEEE subset in that it does not contain large parking lots, parking garages, or major roads. Instead, it contains smaller, closely spaced buildings surrounded by trees, narrower tree-lined streets, and fewer vehicles. In addition to the challenges presented by a low-density point cloud, tree overhang can sometimes occlude or partially occlude vehicles parked along the roadside or in driveways. The ground truth contains 165 polygons outlining the perimeters of the vehicles. The vehicle polygons were created algorithmically from individual vehicle clusters based on the point classes provided.

### 3.3. The DIRSIG Scene

**Figure 3:**
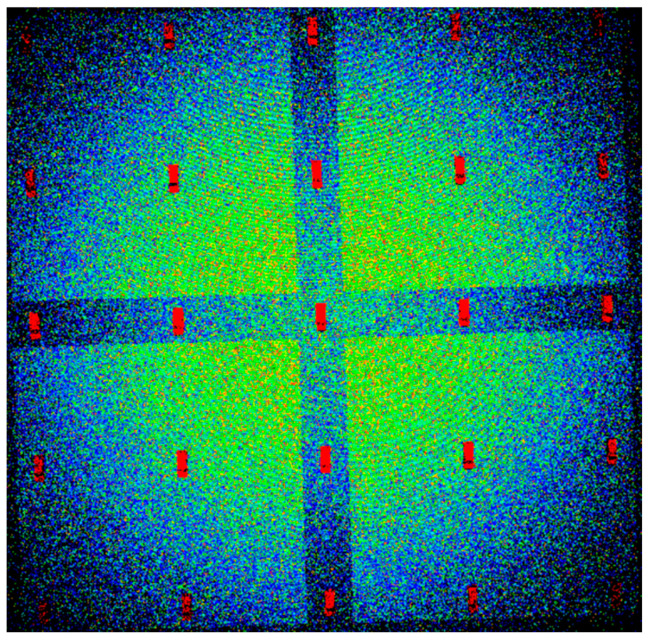
Generated DIRSIG scene showing the ultra-high point density of 31.65 ppsm. The color scale represents elevation from low (blue) to high (red).

**Figure 4:**
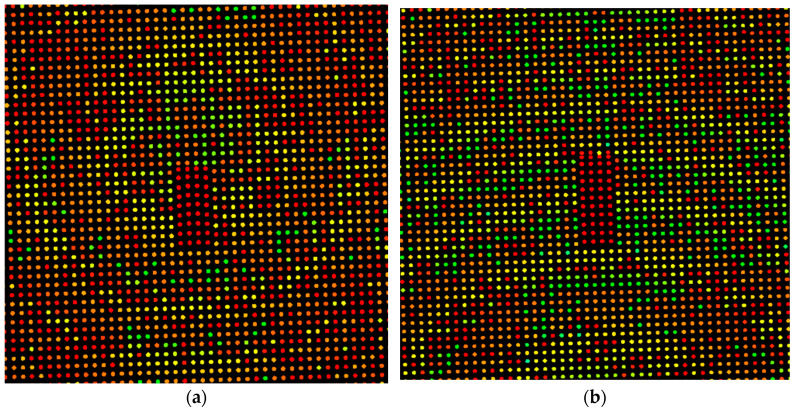

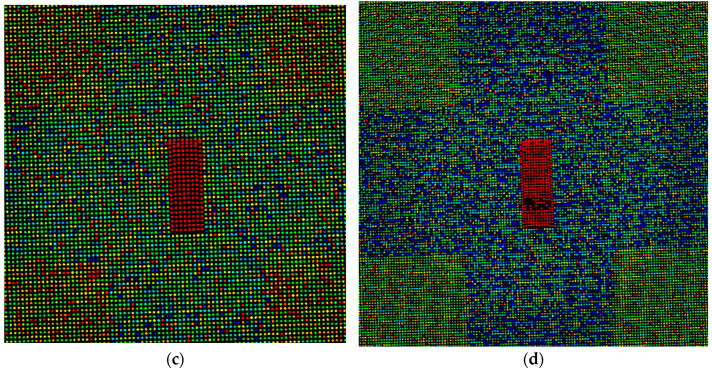
The same car extracted from each of the four scenes with different point densities: (**a**) Base: 3.25 ppsm; (**b**) Low: 5.23 ppsm; (**c**) High: 17.37 ppsm; (**d**) Ultra-High: 31.65 ppsm. The color scale represents elevation from low (blue) to high (red).

The DIRSIG scene differs from the other datasets, because the scene is simulated. DIRSIG is an image generation model that was developed by the Digital Imaging and Remote Sensing Laboratory at the Rochester Institute of Technology [20]. DIRSIG is a powerful tool often used to evaluate imaging system configurations or evaluate algorithms on synthetic data. In this case, DIRSIG was used to generate point clouds of a scene containing 25 identical vehicles. The scene covered a spatial extent of 110 m × 110 m, with an elevation range of 17 m. In total, four-point clouds were generated, each with a different point density. The densities were 3.25, 5.23, 17.37, and 31.65 ppsm. The vehicles were arranged in a grid pattern on a small hill with two roads perpendicular to each other segmenting the scene. Figure 3 shows the entire DIRSIG scene using the ultra-high point density of 31.65 ppsm, while Figure 4 contains a subset of the scene for each point density, emphasizing the number of points on target at each density. The purpose of this dataset is to gauge the performance of the vehicle detection algorithm at different point densities.

## 4. Algorithm Overview

**Figure 5:**
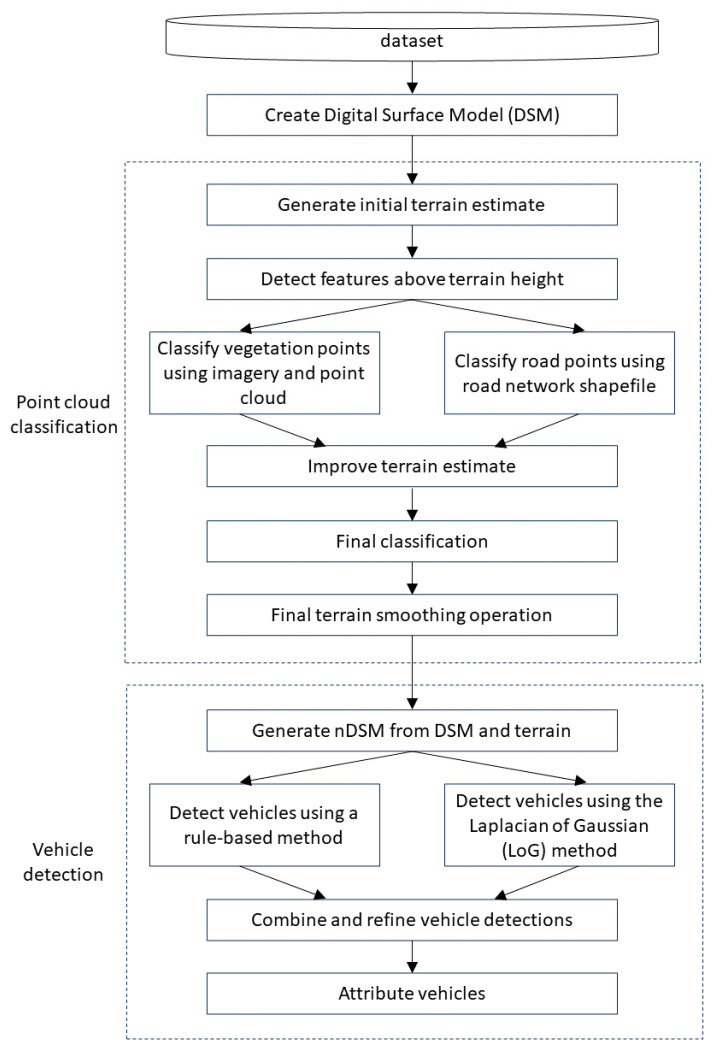
Flowchart outlining the vehicle detection process.

The vehicle detection algorithm is broken down into two main components, as depicted in Figure 5. The first is point cloud classification, which assigns each point in the point cloud a single class label and generates a terrain estimate. The point classes and terrain estimate are then passed into the second module, vehicle detection. The vehicle detections are generated from combining two different detection methods, a rule-based method and a Laplacian of Gaussian method. The vehicles are then attributed with various properties, which provide valuable information during analysis.

### 4.1. Point Cloud Classification

Point cloud classification is generally the first step in any feature extraction method using a point cloud. In the point cloud classification algorithm, only a raw point cloud is necessary to generate classified points. However, additional input data from multispectral imagery (MSI) or road vectors provide significant improvement to the classification accuracy.

Using this particular algorithm, a point can be classified as one of five classes: unassigned, ground, vegetation, building, or road surface. The point classification algorithm includes five main steps, outlined below, but first, the method to generate a digital surface model (DSM) is explained, as the concept is used throughout the algorithm. To generate a DSM, a raster resolution is first automatically selected based on the density of the point cloud, and a grid of empty cells is initialized. Values are assigned to the grid based on the points that fall into each cell. A DSM of minimum elevations (using the lowest point elevation to set the cell value) is ideal for capturing features such as terrain and buildings, whereas a DSM of maximum elevations (using the highest point elevation) is ideal for analyzing features such as tree canopies and power lines.

**Figure 6:**
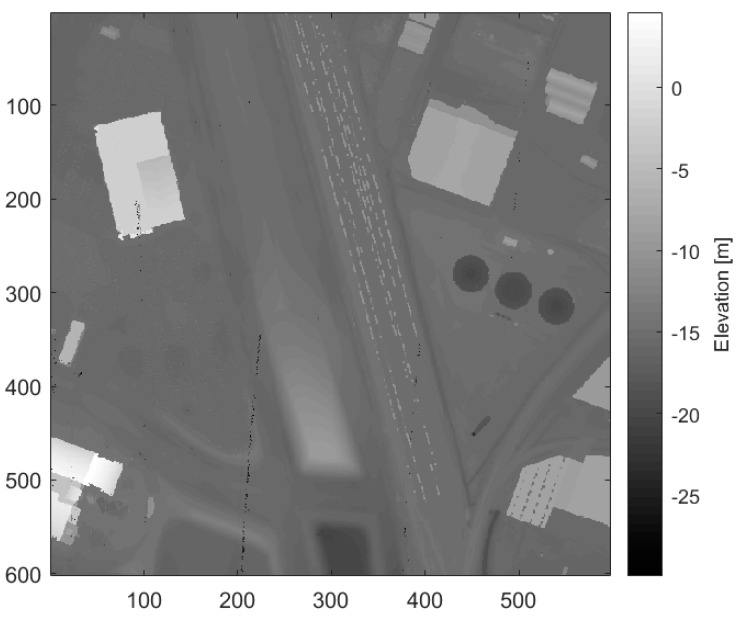
Minimum DSM generated from the IEEE Houston dataset with voids filled and outliers removed (spatial resolution = 1 m).

Since the goal is to generate an accurate terrain estimation, the DSM of minimum elevations (minDSM) is used as the surface model. It is not uncommon for a DSM to have some cells without an assigned value. These cells are the result of voids in the raw point cloud data caused by shadows from a feature (i.e., single viewpoint), absorption of the laser in water, or a gap in the flight path. Small voids are interpolated using a mean filter, while larger voids are filled using a low-resolution terrain estimate. The low-resolution terrain estimate is created from a fifth percentile DSM at a fixed resolution of 50 m. Lastly, an outlier removal process is run on the DSM to remove single cell outliers. For each cell, the outlier removal process computes the difference between that cell and each cell in its local neighborhood. These differences are then compared to the minimum difference considered to be an outlier. If a majority of these differences fall above the threshold, the cell is considered an outlier. This only finds outliers that are greater than their surroundings; a similar method is used to find outliers that are lower than their surroundings. Figure 6 shows the DSM generated for the IEEE Houston dataset.

#### 4.1.1. Generate Initial Terrain Estimates

**Figure 7:**
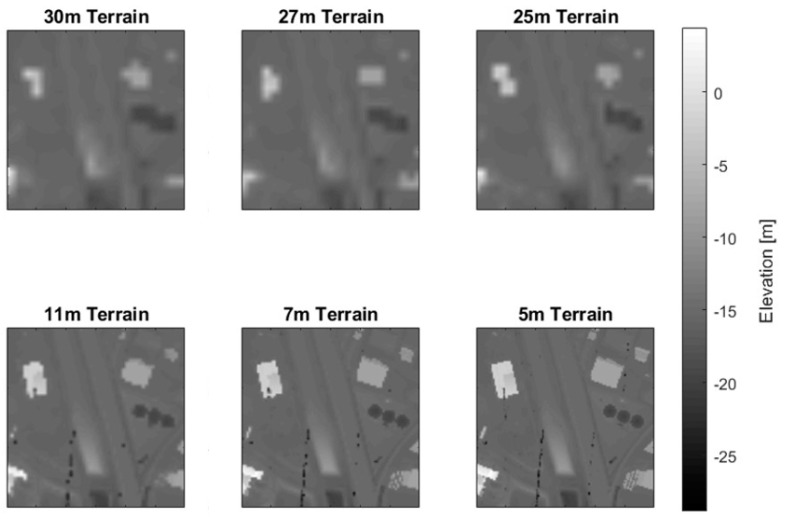
Low- and high-resolution simple terrain rasters from the IEEE Houston dataset created using the fifth percentile of the points in each cell. The spatial extent of the scene is ~600 m × 600 m with a spatial resolution of 1 m.

Using the method for generating a DSM as described above, the fifth percentile statistic is used to generate six terrain estimates: three at a lower resolution and three at a higher resolution. A slight spread in the resolutions for both sets of rasters ensures that a feature edge will not fall directly on a grid line. The different sets of resolutions are used to capture varying levels of detail. Each terrain estimate is created using a fifth percentile DSM at the specified resolution, as shown in Figure 7. A fifth percentile DSM uses the fifth percentile of points, in the *z*-direction, contained in each grid cell.

#### 4.1.2. Identify Features above the Terrain

**Figure 8:**
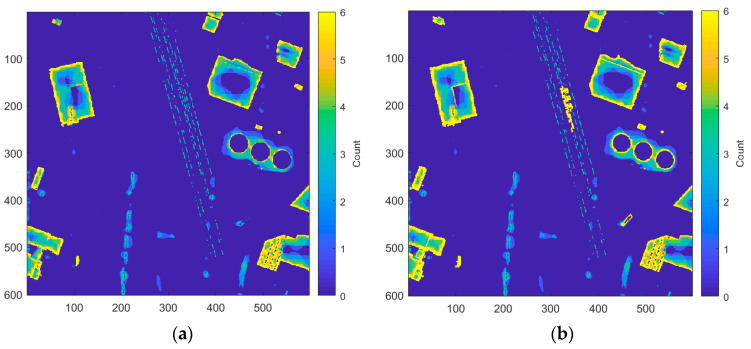
Aggregation of “aboveground” pixels forming a raster of counts using the IEEE Houston dataset. Strong responses on the edge indicate the feature is a building (spatial resolution = 1 m): (**a**) original; (**b**) modified by Laplacian edges.

For each terrain resolution, a pixel is determined to be above the ground if it is at least a minimum height above the terrain estimate. In this case, a minimum aboveground feature height threshold of 2.5 m is used. Once the aboveground points are found at each resolution, they are aggregated to form a raster indicating the number of times each pixel was considered aboveground. The result of this step can be seen in Figure 8a. In this raster, buildings have a strong response along the perimeter that grows weaker towards the center, which separates it from mountains and hills that have an increase in response toward the center. This can be used to remove false positives (mountains and hills) from the non-terrain map by stating that all non-terrain must contain a response from each resolution along the perimeter. First, though, the raster containing the counts of aboveground points must be modified to include missed building detections.

The resulting missed detections of this method appear mainly in urban areas where there tend to be large, multitiered city blocks. In this case, the lower buildings in the city block tend to be pulled into the terrain estimate, since they appear in the lower fifth percentile of the grid cell. This results in the buildings in the DSM not exceeding the threshold to be considered aboveground when compared to the terrain. To incorporate these cases as “non-terrain”, a Laplacian filter is applied to the DSM to identify pixels containing edge content. The edge pixels belonging to a building typically form a hollow contour, while those belonging to trees or noise, for example, typically form a dense blob or line. Using these characteristics, we can create rules to remove these false positives from the Laplacian edge map and keep only buildings. These newly found buildings are then used to boost the raster of “aboveground” counts in locations where there were weak or missed building edges, as shown in Figure 8b.

**Figure 9:**
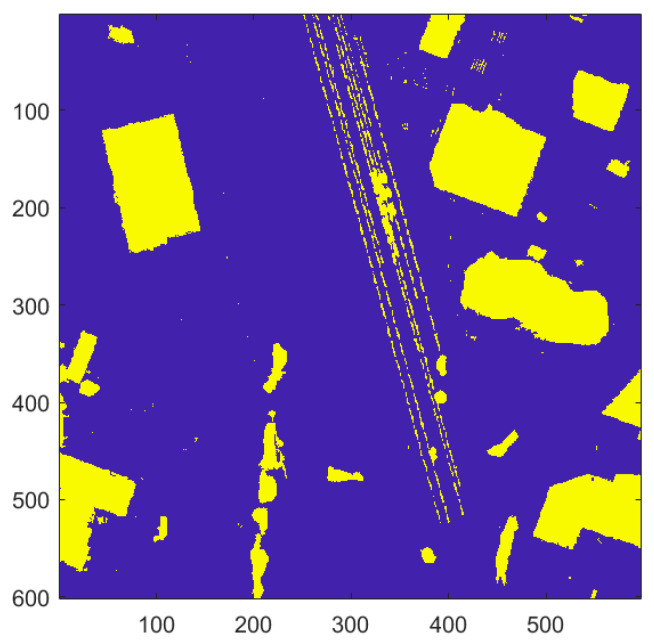
Raster showing the locations of “non-terrain” (yellow) and terrain (blue) for the IEEE Houston dataset (spatial resolution = 1 m).

If a pixel was considered aboveground the majority of the time, it was added to a binary feature map. Relatively small steep hills are often initially misclassified as aboveground because of the similarities in response with buildings. In order to combat this, regions with a low slope are identified using the maximum local slope approach developed by Vosselman [25] and removed from the binary feature map. Finally, morphological operations are applied to clean up the binary raster. The final non-terrain raster appears in Figure 9.

#### 4.1.3. Classifying Points Using Additional Information

Separately from the binary feature map, which is mostly comprised of buildings, the algorithm attempts to identify points belonging to the vegetation and road classes. While classifying road points can only be done if road networks are provided, vegetation points can be classified using MSI imagery, multi-returns from LiDAR point clouds, or both.

**Figure 10:**
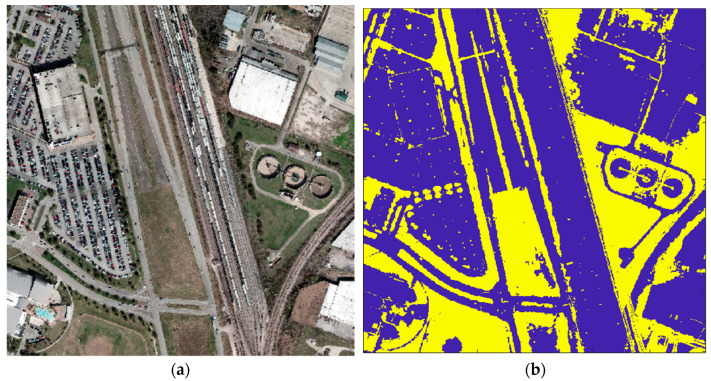
Result of the vegetation indices applied for the IEEE Houston dataset (spatial resolution = 1 m). (**a**) RGB image; (**b**) vegetation map (vegetation in yellow and non-vegetation in blue).

MSI imagery is used to calculate the Normalized Difference Vegetation Index (NDVI), as explained by Jackson and Huete [26]. NDVI values range from −1.0 to +1.0, with sparse vegetation typically ranging from 0.2 to 0.5 and dense vegetation from 0.6 to 0.9. Since NDVI values can vary from scene to scene and depend on the time of year, a scene-adaptive NDVI threshold is computed. Multi-return vegetation is identified by comparing the difference between a minimum and maximum DSM to a slope threshold. These methods yield a vegetation mask, shown in Figure 10, which is added to the binary feature raster.

The RGB vegetation classification method is unique in the fact that it can operate on a MSI image (as long as it contains bands corresponding to the red, green, and blue channels); RGB image; or a colorized point cloud. Numerous RGB vegetation indices exist, and each is designed with a specific application in mind. It is difficult to find an RGB vegetation index that is general and all-inclusive. To overcome this, five different RGB indices are used. If a pixel or point is labeled as vegetation in at least four of the five indices, it is considered to be vegetation.

The first step in classifying roads is to rasterize the road vectors. Then, a map of distances to roads is created. Points that are within three meters of the road vector are initially considered to be road points. False positives are removed by excluding those points that are much higher than the surrounding road points, too dark, or too green. These points are removed due to the likelihood that they are shadows or trees.

#### 4.1.4. Improving the Terrain Estimate

Now that the algorithm has sorted the points into likely classes, we can use these classes to obtain a better terrain estimation. The new terrain raster is initialized by computing the fifth percentile DSM. Features that lie above the terrain are then masked out. The terrain values for these masked regions are obtained through interpolation, resulting in the final terrain estimate.

#### 4.1.5. Final Classification

At this point in the algorithm, it is conjectured that no point concretely belongs to a class. Each point can belong to zero, one, or more classes. This step of the algorithm matches each point to a single class.

Dense vegetation points are often confused for buildings and contained in both the vegetation and building classes. To determine which of the two classes is correct, we compute the variance in the *z*-direction of the region. Since vegetation has a larger variance in height compared to buildings, the regions in question that have a high variance are removed from the building class and maintained in the vegetation class.

Buildings are a direct result of the aboveground raster and maintain the building class label if they are at least building height and have not already been classified as trees.

Points thought to be terrain are compared to the interpolated terrain raster. If a terrain point is significantly above or below the interpolated terrain height or has already been classified as a tree or building, then it is removed from the terrain class.

**Figure 11:**
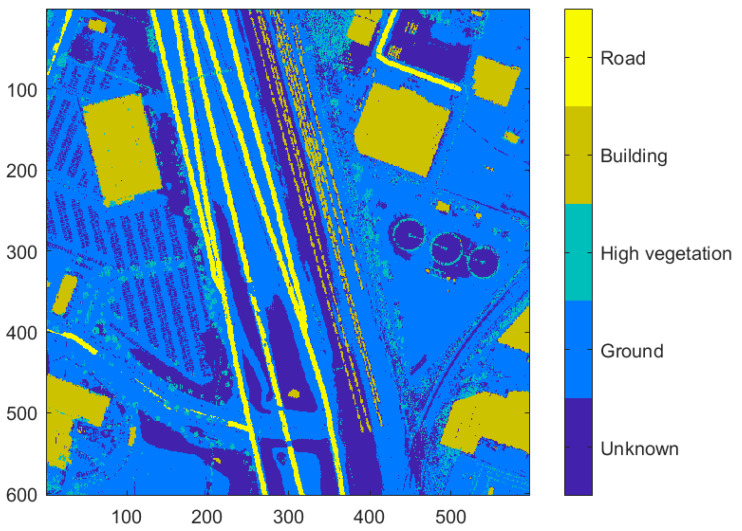
The classified points from the point classification algorithm (spatial resolution = 1 m). The raster is created using the classification label of the point nearest to the pixel center.

Road points maintain the road label. All other points that were not assigned a class assume the “unknown” label. The classified terrain points are passed through one final interpolation and smoothing operation before entering the vehicle detection module. The final classification map is shown below in Figure 11.

### 4.2. Vehicle Detection

This section provides a detailed explanation of the vehicle detection algorithm. It first describes the process of generating a normalized digital surface model (nDSM), which is an integral part of the two vehicle detection methods used. The rule-based approach in Section 4.2.2 and the Laplacian of Gaussian method in Section 4.2.3 are fused in a way that supplement the other to provide a result with relatively few missed detections and false positives. The vehicle detections are then refined and attributed with spatial, spectral, and relational properties.

#### 4.2.1. The Normalized Digital Surface Model

Typically, a nDSM is the result of subtracting the terrain from the DSM, leaving aboveground features such as trees, buildings, and vehicles. This approach works very well, with the exception of vehicles located on the tops of parking garages. In order to obtain a nDSM that includes these vehicles, a variation of the nDSM is used. A surface raster is used for subtraction instead of a terrain raster. This surface raster leaves large surface features, such as buildings, and removes small features, such as vehicles, through a morphological erosion operation. When subtracted from the DSM, only vehicles and other small objects are left.

#### 4.2.2. The Rule-Based Method

The rule-based approach operates on a binary version of the nDSM. Before converting it to a binary raster using a local-adaptive threshold, a morphological opening is applied to the nDSM to remove unwanted bright artifacts in the scene. Regions of the raster that are known to belong to other classes, such as vegetation and buildings, are also removed. Once this preliminary raster is generated, each object is analyzed. If the object exceeds or falls short of a user-defined length, width, or height, the object is removed from the raster. The remaining objects are considered detections and are joined with the Laplacian of Gaussian detections before being refined.

#### 4.2.3. The Laplacian of Gaussian Method

The Laplacian of Gaussian filter (LoG) is commonly used for blob detection or to locate edges in an image. Kong et al. proposed a generalized Laplacian of Gaussian filter (gLoG) for blob detection that is unique due to the lessened number of parameters that need to be tuned [27]. This is the approach used to generate the kernels and apply the gLoG operator for vehicle detection. Before applying the gLoG operator, the nDSM is converted to a binary raster, and locations too high or too low to be vehicle height are masked out according to the user-defined parameters. The kernels are then applied to the binary nDSM, and the stack of responses are then summed per pixel, and the regional maximum is determined. Similar to the rule-based method, objects that do satisfy the user-defined size parameters are removed. These candidates are then combined with those from the rule-based method.

It is possible and rather common when combining vehicle candidates with those from the rule-based method that overlapping or duplicate detections need to be removed. The first step in choosing the best response is to obtain the footprint, or bounding box, of the candidate. Once the bounding box is created, candidates with significant overlap are identified, and a fill factor is used to keep the bounding box with the most fill or vehicle pixels.

#### 4.2.4. Refining Vehicle Detections

Currently, candidate vehicles could be any object with roughly the same length, width, and height as a car. Common false detections often include objects such as small shrubs, despite efforts to remove vegetation. These candidates are further refined by observing unique characteristics about the object that set it apart, such as smoothness and slope.

To evaluate smoothness, a lengthwise profile through the center of the candidate region is extracted and analyzed. Using the standard deviation of the difference in height between adjacent points along the profile line gives the measure of smoothness. If the profile is relatively smooth, the candidate is kept; otherwise, it is removed. This helps to remove detections due to vegetation and noise, among others.

Slope is another way to reduce false alarms. Relative to the ground, vehicles tend to have a slightly negative slope. Using this metric allows for false detections with a significantly steep slope to be removed.

#### 4.2.5. Vehicle Attribution

In order to increase the value of vehicle detections, each vehicle can be further analyzed using a wide gamut of vehicle attributes spanning 3D and spectral properties, as well as relational information and vehicle profiles. Attributing vehicles allows a richer picture of the vehicles in a given location.

**Figure 12:**
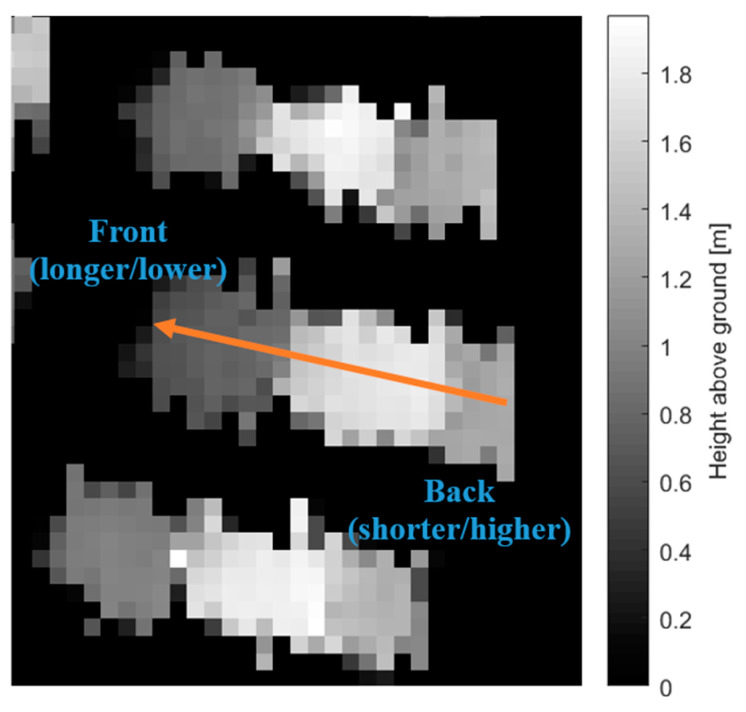
Orientation of a vehicle as determined by its profile indicated by the orange arrow.

Three-dimensional properties are properties that describe the geometry of a detected vehicle in the *x*, *y*, and *z*-directions. These are outlined in Table 1. The orientation is determined from the best-matching gLoG filter, while the length, width, height, easting, and northing are determined from the binary map of the vehicle. The slope is used to determine the heading of the vehicle. The front of a vehicle is generally longer and lower than the back of a vehicle, which creates a slope that can be analyzed to determine the heading. An example is depicted in Figure 12. The vehicle surface points are a list of all points contained within the detection. These vehicle points can be analyzed to determine the vehicle class either manually or by fitting the points to a model using an external algorithm. This feature is particularly useful if there are specific detections of interest or the specific class of each vehicle must be known, such as car, truck, or van.

Spectral properties are properties that capture the response of a detected vehicle from either the MSI imagery or LiDAR data. The intensity values are a direct result of the raw per point intensity values attributed to the point cloud. The NDVI values are derived spectral properties from the MSI imagery. The intensity and NDVI attributes can be used to query and refine detections to obtain a lower false-positive rate, with a slight sacrifice of true detections. These are also shown in Table 1.

**Figure 13:**
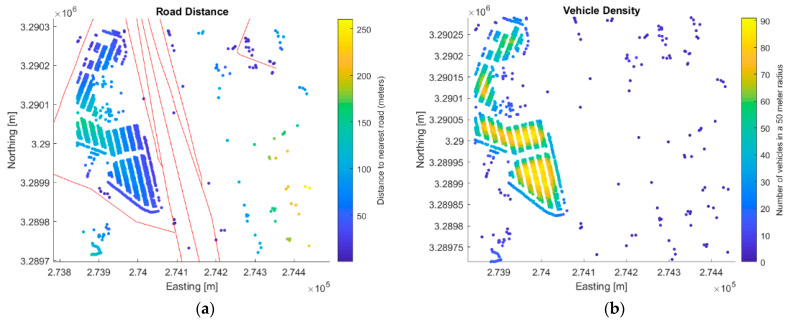
Plots showing two relational attributes: (**a**) the distance from each vehicle to the nearest road; (**b**) the number of vehicles within a 50 m radius. Red lines indicate roads shown in Figure 10 represented as vectors.

Relational attributes describe the relationship that a vehicle has with the surrounding features, including roads and other vehicles. These relational attributes are also outlined in Table 1. The distance to the nearest road could be a useful attribute for an analyst for a variety of reasons. It could eliminate false detections, aid in isolating unique vehicles, identify vehicles within close proximity of a road (in a parking lot, etc.), or identify vehicles in transit. The road distance attribute is only populated if road vectors are provided as input. Figure 13a shows a map indicating the distance of each vehicle to the nearest road. The attributes pertaining to the nearest vehicle (nearest vehicle, nearest orientation, and nearest ID) and vehicle density can be used to generate a hypothesis about the location of the vehicle based on its relationship with others in close proximity. For example, vehicles detected in high-density regions with similar orientations are likely to be located in parking lots. Figure 13b shows the vehicle density of the same scene.

## 5. Evaluation and Results

The goal of this section is to provide detailed results and analysis for the three different datasets outlined in Section 3. This section will review the metrics used and present the results for both the IEEE and ISPRS datasets. The DIRSIG dataset will be used to explain the results and discuss the effect of resolution and point density on the vehicle detections.

### 5.1. Metrics

The analysis in this section is based on the comparison of ground truth vehicles to the detections output by the algorithm. A detection is considered a true positive (TP) if it overlaps the ground truth vehicle by at least 50%; otherwise, it is considered a false positive (FP). A false negative (FN) is a vehicle in the ground truth that has not been detected by the algorithm with at least 50% overlap. Precision and recall provide information about the false-positive rate and missed detection rate, respectively, while the F1 score (defined as the harmonic mean of the precision and recall) balances the two, giving an overall metric.

### 5.2. Results

**Figure 14:**
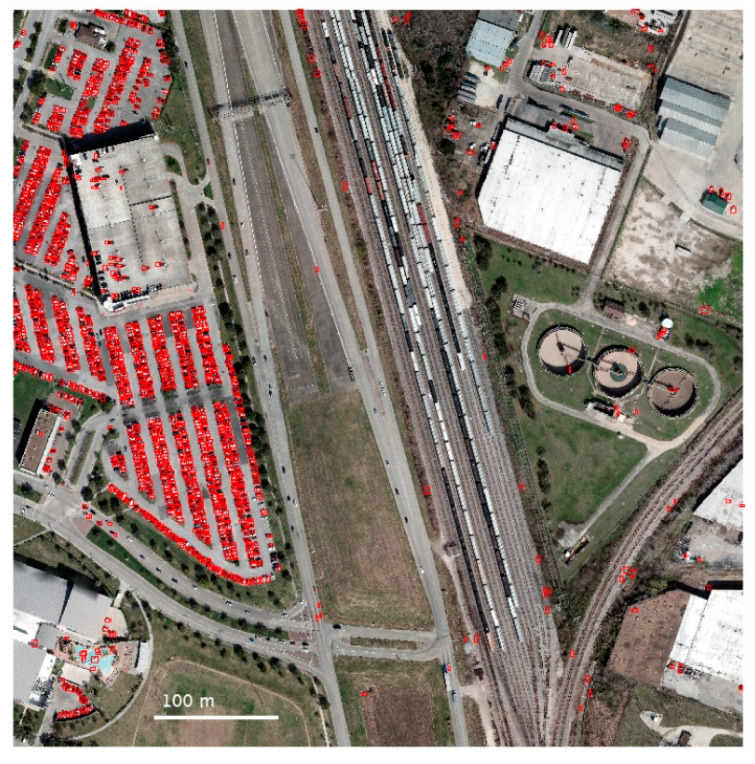
Results from the IEEE dataset with detected vehicles (red) overlaid on the RGB imagery. Moving vehicles on the road were not easily detected due to motion blur in the LiDAR point cloud.

**Figure 15:**
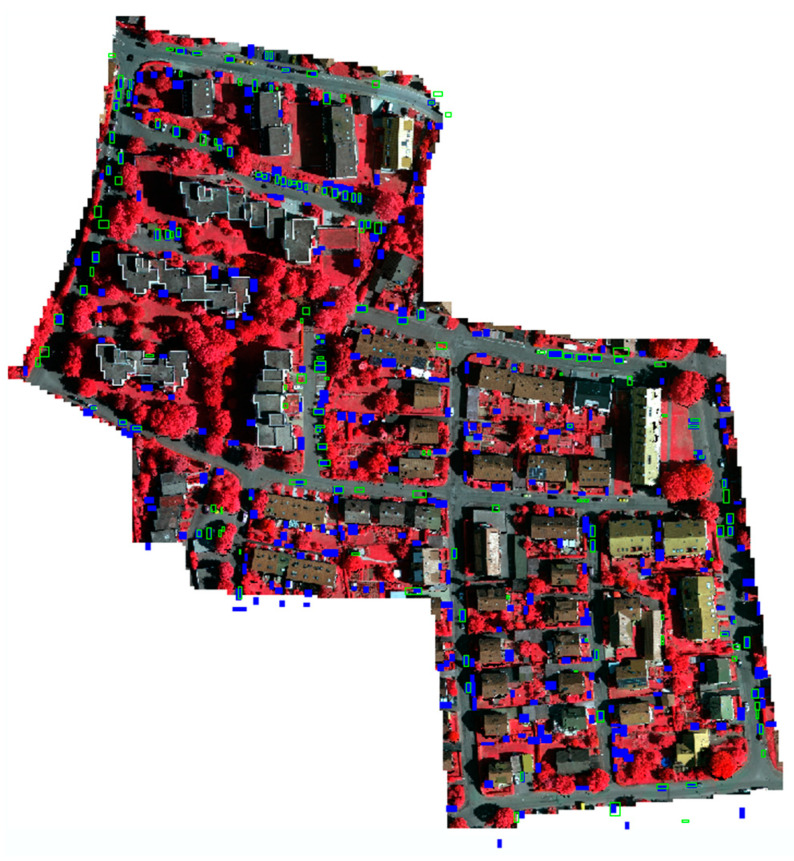
Results from the ISPRS dataset with detected vehicles (solid blue) and ground truth (hollow green) overlaid on the NRG imagery. The cyan border occurs for true positives. Missed vehicles are shown as hollow green rectangles. Red colors represent vegetation. Discrepancies exist between the LiDAR data (on which the detections and ground truth are based) and NRG imagery, since they were not co-collected.

A summary of the results is provided in Table 2. There is a large gap in the performance between the IEEE and ISPRS datasets, especially with precision, indicating a significantly higher false-positive rate for the ISPRS dataset. A visual inspection of the results from the IEEE and ISPRS datasets, in Figure 14 and Figure 15, respectively, shows that many false positives due to buildings and other structures are virtually eliminated, but that the algorithm is also susceptible to detecting any object with the same length, width, and height as a vehicle.

In the IEEE dataset, these types of false positives commonly include leaf-off or unhealthy vegetation, as well as industrial objects such as stacks of railroad ties, collections of barrels, piles of tires and scrap metal, and dumpsters. For the ISPRS dataset, these false positives tend to be vegetation, despite efforts to remove vegetation using the NDVI and multi-returns from the point cloud. This could be due to a few reasons. The first is that many of the false positives due to vegetation in this scene are hedges. LiDAR systems do not penetrate dense vegetation very well and therefore produce few to no multi-returns in these regions. The second is due to the fact that the resolution of the imagery (from which the NDVI is calculated) is much greater than the resolution of the point cloud and, consequently, the nDSM. The imagery is therefore downsampled, and a significant amount of information is lost, creating a noisy image. As a result, more artifacts are introduced when masking out vegetation in the binary map. Each artifact is only composed of a few pixels, but with such a low resolution, each vehicle is also only represented by a few pixels, making vegetation artifacts confusing for vehicle detection.

### 5.3. DIRSIG Point Density Study

Given the difference in results between the IEEE and ISPRS datasets, with the main difference being point density, a study was performed using simulated point clouds to determine the effect of point density on vehicle detection. The point density of the IEEE dataset (~33 ppsm) is represented closely by the DIRSIG Ultra-High density, while the point density of the ISPRS dataset (~4 ppsm) falls between the DIRSIG Base and DIRSIG Low densities. From Table 2, the recall for the IEEE dataset is 0.92, while the ISPRS dataset has a recall of 0.62. This closely matches the trend shown in Table 3, where the likelihood of detecting vehicles using this algorithm decreases with the point density and helps explain the significant differences seen between the two datasets.

### 5.4. Computing and Timing Considerations

The end-to-end process ran in under five minutes on a standard machine for the IEEE dataset and was not optimized for runtime. This approach is not a machine learning technique and does not require a lot of computation power.

Typical timing results on the IEEE dataset are shown below.

- Reading the point cloud data: 11 s
- Classifying the point cloud: 29 s
- Computing the initial terrain estimate: 61 s
- Finding buildings: 45 s
- Rerun terrain with updated labels: 52 s
- Run the car detector: 39 s
- Obtain the car boundaries: 47 s
- Approximate total time: 284 s

## 6. Conclusions and Future Work

### 6.1. Conclusions

In this paper, a classical approach to vehicle detection was taken that relies on neither training data nor models. Instead, it extracts vehicles using a rule-based approach in combination with data fusion from LiDAR point clouds, RGB imagery, and road networks. The algorithm achieved a F1 score of 0.92 on the 2018 IEEE dataset, with room for several promising improvements. Point density was found to have a direct and significant impact on the success of the algorithm, as found with the ISPRS dataset and shown in the DIRSIG point density experiment. This indicates that fusing detection methods, especially ones that utilize different sensing modalities, could greatly benefit the current implementation.

### 6.2. Future Work

There are several new capabilities and features that can be added to improve the algorithm and increase its usability. One of these improvements lies in identifying the type of vehicle (car, truck, van, etc.) from a set of existing 3D models. There is also great benefit in exploring other classical approaches to vehicle detection. Currently, the approach combines two different algorithms that both base their detections off of the nDSM and are searching for objects with dimensions similar to those of a vehicle. Additional information such as color composition, color edge detection, shadow features, and the spectral comparison of an object to its local background are all ways in which imagery can be exploited to improve vehicle detections. Basing detections off of several key characteristics, such as physical dimensions and spectral properties, instead of a single characteristic has the potential to increase confidence in detections and overcome the limitations of any individual method. Furthermore, the comparison of our approach to other existing methods for vehicle detection using data fusion is a topic of our future research in order to better understand and assess the efficacy of our approach.

## Figures and Tables

**Table 1:** Vehicle attributes.

Attribute	Type	Definition	Unit
Vehicle ID	-	unique ID of the vehicle	unitless
Length	3D	length of the semi-major axis	meters
Width	3D	length of the semi-minor axis	meters
Height	3D	height of the vehicle at the centroid	meters
Orientation	3D	orientation of the longitudinal axis of the vehicle from 0° ≤ *θ* < 180°	degrees
Heading	3D	traveling direction of the vehicle relative to true north, from a 0° to 360°	degrees
Slope	3D	slope along the semi-major axis	unitless
Easting	3D	eastward measure of the centroid in UTM coordinates	meters
Northing	3D	northward measure of the centroid in UTM coordinates	meters
Vehicle Surface Points	3D	(*x*, *y*, *z*) coordinates for each point contained in the vehicle bounds	meters
Intensity	spectral	strength of the return pulse	varies by sensor
NDVI	spectral	quantifying index for vegetation	unitless
Road Distance	relational	distance to the nearest road	meters
Nearest Vehicle	relational	distance to the nearest vehicle	meters
Nearest Orientation	relational	orientation of the nearest vehicle	degrees
Nearest ID	relational	ID of the nearest vehicle	unitless
Vehicle Density	relational	number of vehicle detections in a 50 m radius per unit area	m^−2^

**Table 2:** Results for the IEEE and ISPRS datasets.

Dataset	Total Vehicles	TP	FP	FN	Recall	Precision	F1 Score
2018 IEEE	1476	1360	112	116	0.9239	0.9214	0.9226
ISPRS	165	102	239	63	0.6182	0.2991	0.4032

**Table 3:** Results of the DIRSIG point density study.

Dataset	Point Density (ppsm)	Total Vehicles	TP	FN	Recall
DIRSIG Base	3	25	14	11	0.56
DIRSIG Low	5	25	17	8	0.68
DIRSIG High	17	25	19	6	0.76
DIRSIG Ultra-High	32	25	23	2	0.92

## Data Availability

Not applicable.
